# Everyday postures in idiopathic scoliosis: is there any correlation with curve morphology?

**DOI:** 10.1186/1748-7161-8-S1-O6

**Published:** 2013-06-03

**Authors:** A Negrini, E Pasini, M Romano, S Donzelli, F Zaina, S Negrini

**Affiliations:** 1 ISICO (Italian Scientific Spine Institute), Milan, Italy; 2 University of Brescia, Brescia, Italy; 3 IRCCS Don Gnocchi, Milan, Italy

## Background

Postural control is considered important for hyperkyphosis treatment, but it has been defined of mean importance in the SOSORT Consensus on aims of idiopathic scoliosis (IS) treatment [1,2]. Nevertheless, no studies can be found on the usual everyday postures (UEP) of IS patients.

## Aim

To verify if IS patients adopt specific asymmetric UEP.

## Methods

Through parents and scoliosis experts’ consultation, we developed and validated a questionnaire for parents evaluating 7 UEP. Inclusion criteria were IS and age between 6 and 18 years. We collected 635 questionnaires from all IS patients coming to our Institute between September and November 2011 (n=435, response rate 98.5%) and through specific emails (n=199, response rate 15.7%). Since there were no differences between these two groups in gender, age, scoliosis parameters and answers, we evaluated all questionnaires together. We had IS group (ISG: curves >10°; n=462) and controls (CG: curves <10°; n=173). We divided ISG into three pairs of subgroups:

L: lumbar or thoraco-lumbar curve: left (LL-SG n=65); right (RL-SG n=56)

T: thoracic curve: right (RT-SG n=79); left (LT-SG n=11)

DC: double curves: left L right T (LRDC-SG: n=215); right L left T (RLDC-SG n=36)

We compared ISG and all subgroups to CG, and each subgroup to its matched pair (e.g. LL-SG vs RL-SG). All answers were converted as follows: one side: +1; the other side: -1; no preference: 0. Maintaining only the UEP with statistical differences, and checking for the preferred direction, we developed three index of symmetry (IoS) (one per pair of subgroups). Finally, we checked correlations between Cobb degrees, UEPs and IoS.

## Results

In L subgroups, we found one UEP statistically different between the matched pairs and 2 from CG; in the T subgroups the differences were 2 and 2 respectively; in the DC subgroups, only LRDC-SG had 3 postures different from CG. The calculated IoS were significantly different in the the L and T subgroups, but not in DC. There were no statistical correlations with Cobb degrees.

## Conclusions

IS patients have preferred UEP, mainly in the case of single curves; postural control strategies should be considered in future rehabilitation protocols.
